# Management of chest pain: exploring the views and experiences of chiropractors and medical practitioners in a focus group interview

**DOI:** 10.1186/1746-1340-13-18

**Published:** 2005-09-02

**Authors:** Monica Smith, Dana J Lawrence, Robert M Rowell

**Affiliations:** 1 Palmer Center for Chiropractic Research, Palmer College of Chiropractic, 741 Brady Street, Davenport, IA 52803, USA

**Keywords:** Chest Pain, Chiropractic, Medical Education, Coordination of Care

## Abstract

**Background:**

We report on a multidisciplinary focus group project related to the appropriate care of chiropractic patients who present with chest pain. The prevalence and clinical management, both diagnosis and treatment, of musculoskeletal chest pain in ambulatory medical settings, was explored as the second dimension of the focus group project reported here.

**Methods:**

This project collected observational data from a multidisciplinary focus group composed of both chiropractic and medical professionals. The goals of the focus group were to explore the attitudes and experiences of medical and chiropractic clinicians regarding their patients with chest pain who receive care from both medical and chiropractic providers, to identify important clinical or research questions that may inform the development of 'best practices' for coordinating or managing care of chest pain patients between medical and chiropractic providers, to identify important clinical or research questions regarding the diagnosis and treatment of chest pain of musculoskeletal origin, to explore various methods that might be used to answer those questions, and to discuss the feasibility of conducting or coordinating a multidisciplinary research effort along this line of inquiry. The convenience-sample of five focus group participants included two chiropractors, two medical cardiologists, and one dual-degreed chiropractor/medical physician. The focus group was audiotaped and transcripts were prepared of the focus group interaction. Content analysis of the focus group transcripts were performed to identify key themes and concepts, using categories of narratives.

**Results:**

Six key themes emerged from the analysis of the focus group interaction, including issues surrounding (1) Diagnosis; (2) Treatment and prognosis; (3) Chest pain as a chronic, multifactorial, or comorbid condition; (4) Inter-professional coordination of care; (5) Best practices and standardization of care; and (6) Training and education.

**Conclusion:**

This study carries implications for chiropractic clinical training relative to enhancing diagnostic competencies in chest pain, as well as the need to ascertain and improve those skills, competencies, and standards for referrals and sharing of clinical information that may improve cross-disciplinary coordination of care for chest pain patients.

## Background

While the main focus of chiropractic care centers on treatment of musculoskeletal disorders, chiropractors serve as first point of contact with the health care system for patients presenting with a broad range of conditions [1]. As a portal-of-entry healthcare provider in a primary ambulatory setting, the professional responsibilities of the practicing chiropractor include proper assessment, documentation, and treatment of chest pain/discomfort cases, and appropriate and timely referral of chest pain patients as needed.

An extensive body of primary empirical literature addresses patient management protocols (differential diagnosis and diagnostic/treatment algorithms) for patients presenting with chest pain, primarily focusing on cardiopulmonary, gastroesophageal/gastrointestinal, and psychological conditions causing chest symptoms [2-17]. These etiologic sources are ruled out as the cause for many chest pain sufferers, and such patients essentially 'fall out of the algorithm' with ongoing chest pain that remains undiagnosed, untreated, and unresolved.

A small but growing body of literature estimates the presumed prevalence of musculoskeletal chest pain in medical settings at 20–50% [14-18], and reflects a growing awareness that musculoskeletal causes remain largely unexplored as potential sources of chest pain, particularly for chronic or recurrent chest pain that remains undiagnosed and unresolved.

The Cochrane Database of Systematic Reviews (CDSR), containing completed reviews carried out by the Cochrane Collaboration , contains only one citation for chest pain, not musculoskeletal [19]. The Database of Abstracts of Reviews of Effects (DARE), maintained by the NHS Centres for Reviews and Dissemination and linked to the Cochrane Library , includes a number of reviews that focus on comparing various clinical diagnostic test strategies for cardio-related chest pain [20-29], as well as numerous organizational-level studies examining the clinical safety and cost-effectiveness of shifting cardio-related chest pain evaluation units from hospital inpatient to hospital outpatient settings [30-44]. Other review articles returned in the DARE search confirms our impression that current medical approaches to diagnosing, treating, or managing non-specific or non-cardiac chest pain focus principally on psychological and gastroesophageal/gastrointestinal causes and essentially ignore the potential for musculoskeletal etiologies [45-51].

We report on a multidisciplinary focus group project, one aspect of which specifically addressed issues related to the appropriate care of chiropractic patients who present with chest pain, whether as a main presenting complaint or as a co-morbid condition. The prevalence and clinical management, both diagnosis and treatment, of musculoskeletal chest pain in ambulatory medical settings, was explored as the second dimension of the focus group project reported here. The objective was to gain insight into the care and management of patients with musculoskeletal chest pain as experienced by both those with chiropractic training, medical training or combined training.

## Methods

### Data collection

#### Focus Group

This project collected observational data from a multidisciplinary focus group composed of both chiropractic and medical professionals. The goals of the focus group were to explore the attitudes and experiences of medical and chiropractic clinicians regarding their patients with chest pain who receive care from both medical and chiropractic providers, to identify important clinical or research questions that may inform the development of 'best practices' for coordinating or managing care of chest pain patients between medical and chiropractic providers, to identify important clinical or research questions regarding the diagnosis and treatment of chest pain of musculoskeletal origin, to explore various methods that might be used to answer those questions, and to discuss the feasibility of conducting or coordinating a multidisciplinary research effort along this line of inquiry.

### Population, setting, timeframe

The convenience-sample of five focus group participants included two chiropractors, two medical cardiologists, and one dual-degreed chiropractic/medical physician. The focus group was conducted in early 2004 at the offices of the medical cardiologists.

### Support documents/instruments

The questions posed to focus group participants are provided in Additional file 1. Aside from presenting the semi-structured questions, running the audio recorders, and ensuring that all questions were addressed within the time allotted for the focus group meeting, the facilitator's role during the focus groups session was intentionally minimalized in order to enhance the authenticity of the observations offered by focus group participants.

### Human subjects

The Institutional Review Board of Palmer College approved this study of human subjects, including the Informed Consent document signed by all Focus Group participants. To protect subject confidentiality, subject records (i.e., signed Informed Consents and verbatim unblinded master transcript) were maintained in a locked file cabinet. The final 'blinded' transcript (all subject identifiers removed) was used during the content analysis, which was performed by all three investigators, although two of the study investigators were also present during the focus group.

### Data management and analysis

#### Focus Group Qualitative Analyses

The focus group was audiotaped and transcripts were prepared of the focus group interaction. Content analysis of the focus group transcripts were performed to identify key themes and concepts, using categories of narratives. All three authors analyzed complete transcripts and developed independent lists of overall themes and concepts subsumed within the general themes. Once completed, the investigators came together to collapse their lists of themes into one set of themes as reached via consensus. This process involved examining themes for commonality, classifying them for uniformity, and then reaching agreement on the final list of six key themes. Once the themes were set and subordinate concepts identified, each investigator looked for quotes and comments which exemplified those themes and concepts (which are presented in the Results, below)

As a methodological 'cross-check', the investigative group's consensus process confirmed observations drawn from each investigator's independent analysis of the transcripts, which strengthened the validity and reliability of the study findings reported from this qualitative research [52,53]. It is important to note that this research is an exploration into the specifics of the convenience sample drawn for the project; therefore, generalizability is not a significant consideration in this study.

## Results

Six key themes emerged from the analysis of the focus group interaction, including issues surrounding (1) Diagnosis; (2) Treatment and prognosis; (3) Chest pain as a chronic, multifactorial, or comorbid condition; (4) Inter-professional coordination of care; (5) Best practices and standardization of care; and (6) Training and education. These thematic issues are summarized below, and key excerpts from the focus group transcript exemplifying these thematic issues are included in Additional file 2.

### (1) Diagnosis

Participants reported that a good history and physical exam are essential and important to good diagnosis, that a history should include all prior care received for that condition, that records of prior care should be obtained directly from the source provider, and that history, exam, and differential diagnosis are central to the provision of portal-of-entry primary care as well as secondary specialty care. They noted that diagnostic uncertainty, complexity, and discriminant variability are characteristic of chest pain assessment and diagnostic tests, that the inherently high risk of chest pain determines the order of differential workup and the path of diagnostic referral care (e.g., rule out cardiac and other major medical conditions first), and that musculoskeletal chest pain is principally a diagnosis by exclusion. Anecdotal experience of both chiropractic and medical cardiology focus group participants confirms reports in the literature of a high prevalence of suspected musculoskeletal chest pain in ambulatory practice settings.

### (2) Treatment and prognosis

Chiropractic participants reported anecdotal evidence (their personal practice experience) of the effectiveness of manual/manipulative approaches to resolve chest pain of suspected musculoskeletal origin. Chiropractic and medical participants both noted lack of formal clinical studies examining effectiveness of manual/manipulative approaches to manage (diagnose and treat) musculoskeletal chest pain, and lack of evidence supporting effectiveness for medical drug interventions for musculoskeletal chest pain (e.g., oral nonsteroidal anti-inflammatory drugs or steroid injections into chest wall), and that it is unknown to what extent drug interventions are prescribed for such conditions in actual current medical practice (generalist or specialist). The agreed that both effectiveness and safety concerns should direct the appropriateness and order of trying various clinical approaches to resolve musculoskeletal chest pain in a given patient, and that a better understanding of the etiology of musculoskeletal chest pain condition(s) would also help discriminate between different conditions and guide the search for identifying effective interventions for a given condition. Natural history or prognosis of treated versus untreated acute or chronic musculoskeletal chest pain is also unknown.

### (3) Chest pain as a chronic, multifactorial, or comorbid condition

It is unknown to what extent chronic, unresolved chest pain may represent undiagnosed musculoskeletal chest pain, or to what extent patients with undiagnosed and unresolved musculoskeletal chest pain are perhaps being misclassified as psychological or psychiatric cases. The participants commented that chronic recalcitrant chest pain is associated with high resource use and unsatisfied, distressed patients, that it is unknown to what extent early manual/manipulative intervention in acute musculoskeletal chest pain may prevent development of chronic musculoskeletal chest pain, that chronic musculoskeletal chest pain may raise patient care issues similar to other chronic conditions (i.e., providers and patients may manage some chronic conditions, rather than resolve them), and that the diagnostic and treatment considerations are further complicated when musculoskeletal chest pain and non-musculoskeletal chest pain may exist together as related or unrelated comorbidities. Finally, they noted that with a higher likelihood of degenerative musculoskeletal disorders in older patients and also higher likelihood of visceral (cardiopulmonary or gastrointestinal) disorders in older patients, chest pain in older patients therefore may be more likely of multi-factorial etiology and more likely associated with comorbidities.

### (4) Inter-professional coordination of care

Participants reported that referrals should be based on evidence of efficacy/effectiveness for a given condition such as musculoskeletal chest pain, that the path of referral for chest pain will depend on the nature of the condition and the urgency of the situation, that the point of referral may depend on the familiarity or relationship between the providers, and that the nature of the referral (e.g., amount and type of information accompanying the referral) may depend on the nature of the condition, whether the referral is for reasons of diagnosis and/or treatment, the preference of the provider, and the relationship between the providers. Medical specialists (e.g., cardiology) who receive referrals from primary medical practitioners will most typically return the patient to the primary medical practitioner rather than referring them elsewhere, although this also may depend on the nature of the condition and the relationship between the specialty and primary medical practitioner.

Participants felt that patients with co-morbidities (e.g., having both musculoskeletal and non-musculoskeletal chest pain) may be more likely to receive concurrent care from more than one provider, that providers can pro-actively improve interprofessional relationships by being diligent about sharing pertinent information and reports during referrals. Participants felt that educating other providers about available evidence, recognizing and addressing issues of professional boundary protection (often referred to as 'turf'), and that patients' direct experience (with successful or unsuccessful treatment outcome) and their preferences will also impact provider perceptions and interprofessional relationships.

### (5) Best practices and standardization of care

Participants reported that standardizing care within professions may facilitate opportunities for interprofessional referrals, that guidelines and care standards are an issue for all professions, that interactions between providers and professions (e.g. referrals) may also be standardized, and that 'best practices' for coordinating musculoskeletal chest pain care would center on the role of primary medical practitioners rather than specialist medical practitioners.

### (6) Training/Education

Competencies in exam, diagnostic, and clinical decision-making skills for chest pain were raised as issues for, and by, both chiropractors and medical practitioners. Medical practitioners' perception, familiarity and comfort with chiropractors' diagnostic skills largely comes via direct exposure in postgraduate practice (exchanging clinical reports, etc.) rather than during their medical training. Participants commented that there is a perception that medical education/training is more standardized than chiropractic, and a perception that medical practice is more consistent with medical training (i.e., chiropractors' clinical practice may be more likely to deviate from what they were taught). A comment was made that medical training includes developing skills/competencies in referral practices (e.g., standardized referral forms are used in medical academic practice and teaching clinics).

## Discussion

The focus group dialogue suggested several implications for current and future chiropractic practice, undergraduate and post-professional chiropractic education and clinical training, research, and professional organization or policy. These implications for practice, education, research, and policy are summarized below along with our recommendations.

### Clinical practice

With all portal-of-entry providers such as chiropractors, the responsibility to diagnose chest pain is vital. The focus group touched on this point several times. In order to arrive at a diagnosis for chest pain, or any other condition, they stressed the importance of first taking a good history and then performing a thorough examination. The diagnosis of chest pain, however, is complicated and requires excellent diagnostic skills. The focus group (both the chiropractors and the medical practitioners) expressed a concern over the ability of chiropractors to accurately diagnose chest pain. In order for chiropractors to have a role in managing chest pain from the point of entry, they must acquire and demonstrate competence in diagnosing the complaint.

Chest pain can have a multitude of etiologies, involves an inherently high level of diagnostic uncertainty, and diagnostic algorithms are complex. The clinician's most immediate concern is ruling out emergent versus non-emergent conditions [54-58]. The focus group participants were unified in voicing the need for rapid diagnosis and management for cardiopulmonary conditions such as myocardial infarction and pulmonary embolism, among others. They also pointed out that the clinical picture of chest pain can be complicated by simultaneous etiologies. For example, one of the medical doctors noted that in his own practice he saw patients with cardiac disease and chest wall tenderness. This sentiment was echoed by one of the chiropractors who noted that simply palpating a patient's chest wall and finding tenderness does not rule out cardiac or other life threatening causes of chest pain. Therefore, a full chest pain work up must include evaluations for cardiac, pulmonary, gastrointestinal, musculoskeletal and psychological causes of chest pain.

Once life threatening causes of chest pain have either been ruled out or managed, other possible etiologies may be investigated. The focus group expressed concern that musculoskeletal chest pain may be either missed or misdiagnosed as psychological in nature. The misdiagnosis of musculoskeletal chest pain as psychological could cause much distress, cost, and unnecessary suffering for patients. It is important, therefore, to investigate efficient and accurate diagnostic strategies for this complaint.

Participants in the focus group commented that musculoskeletal chest pain is common in their practices, both chiropractic and medical. This is consistent with reports in the literature that 20%–50% of chest pain presentations in ambulatory settings may be musculoskeletal [14-18]. Management of chest pain of cardiac or gastrointestinal origin is much more standardized than musculoskeletal chest pain. Appropriate protocols and treatment algorithms do not exist for musculoskeletal chest pain. Manipulation, physiological therapeutics, injections, analgesics, and other treatments may be employed, but none have been extensively investigated.

The opportunity for cross-disciplinary coordination of care for chest pain certainly exists. Unfortunately, effective referral pathways do not exist. Chest pain in medical practice is often diagnosed by cardiologists who then send patients back to their referring clinicians, most likely a primary care medical physician. Therefore, it is important to ascertain and possibly improve those skills, competencies, and standards for referrals and sharing of clinical information that may improve current and future cross-disciplinary coordination of care for chest pain patients.

### Clinical and health services research

It is apparent that there is insufficient scientific evidence to guide clinical practice and decision making for musculoskeletal chest pain. The focus group chiropractic participants largely reported only personal anecdotal evidence for the effectiveness of manipulative interventions for chest pain of suspected musculoskeletal origin, and both the chiropractors and medical practitioners commented upon this lack of evidence, both in terms of therapy as well as for diagnosis. Similarly, there is a concomitant lack of evidence supporting the chemotherapeutic interventions used by medical doctors for suspected musculoskeletal chest pain.

Thus, there is much that is not known. Research questions worth investigating include:

• What is the incidence and prevalence of musculoskeletal chest pain in chiropractic clinical practice?

• What is the incidence and prevalence of musculoskeletal chest pain in specialist cardiologist practice? In general medical practice?

• What percentage of chiropractors treat musculoskeletal chest pain compared to those who refer out for care?

• How effective is manipulation for treating musculoskeletal chest pain?

• What other modalities do chiropractors use during such treatments?

• What diagnostic methods are used for determining the presence of musculoskeletal chest pain? What is the reliability, validity, sensitivity and specificity of each test?

• What are the costs involved in treating such cases?

• What interdisciplinary models exist with regard to developing coordinated-care protocols for diagnosis and treatment of acute musculoskeletal chest pain? For long-term management of chronic or recurrent musculoskeletal chest pain?

• Do incidence and prevalence rates vary geographically or by setting?

One challenge relative to chiropractic research is that funding sources are limited and few opportunities exist. So, this presents a conundrum, in that more research is needed but the greatest amount of resources (both funding, and limited research workforce) are directed toward conditions which are already well established with regard to chiropractic research: low back pain, neck pain, and headache.

A Search using the key terms "Chiropractic" and "Musculoskeletal Chest Pain" on PubMed yielded only three papers, two of which had no real pertinence to the issue at hand. The third paper was a case report that looked at using a specific chiropractic adjusting procedure for managing chronic chest pain [59]. There were no randomized trials found in the literature. Shifting the search to the terms "Medicine" and "Musculoskeletal Chest Pain" improved the yield to just nine papers, one of which was a repeat from the chiropractic search, and several of which were tangential to this issue. Obviously, this is an area needing far more research.

As a first pass at documenting and better understanding this problem area, it would be useful to survey the chiropractic profession to quantify rates of musculoskeletal and non-musculoskeletal chest pain presentations in clinical practice, whether as a chief complaint, or as a related or unrelated comorbid condition. The incidence rate of chest pain presentations to chiropractic teaching clinics has been estimated to range from 1% to 7% [60,61], but rates in a typical chiropractic practice are essentially unknown. In surveying chiropractors regionally or nationally, it would also be worthwhile to compare incidence and prevalence rates in rural versus non-rural chiropractic practice, given that chiropractors located in rural or underserved areas may be more likely to serve as the patient's first contact with the health care system, or to function as their patient's main or usual source of care in a broader generalist capacity compared to chiropractors in other areas [62,63]. Chiropractors serving as a first contact or portal-of-entry in a primary care setting may be more likely to see chest pain cases presenting earlier during an episode of care-seeking, or more likely as a generalist to serve as a main source of care overall for an entire chest pain episode. Along that same line, a comparison of rates in chiropractic versus medical practice may also provide insight as to potential implications for management and co-management of these conditions and patients in the primary care setting.

It is important to note that such research should of necessity be collaborative and interdisciplinary. As noted, funding opportunities within chiropractic are limited, yet chiropractors are working in collaboration with medical physicians in a variety of settings. Following case reports and case series which suggest a role for manipulative intervention in musculoskeletal chest pain, the next step would be to devise collaborative research within medical settings, acknowledging that this is likely the best location to obtain participants for research. A multi-disciplinary practice-based research effort could provide a foundation for conducting the requisite feasibility studies and generating the necessary preliminary clinical data and methods (e.g., developing protocols and establishing reliability of procedures), that can then guide and justify more extensive, more rigorous, controlled preclinical and clinical trial work along this line of inquiry.

### Education and educational research

What becomes obvious is that a lack of research has impact and implications for the education of both chiropractors and medical physicians with regard to managing chest pain of musculoskeletal origin. This is also the case with inter-professional collaboration and referral. One comment made by a participant (the dual qualified chiropractor/medical practitioner) was that most medical physicians do not know enough about the training of chiropractors, and do not know about chiropractic diagnostic acumen.

In responding to the question asked by one participant as to how relations between chiropractors and medical practitioners might change, one of the chiropractic physicians answered "better education" and noted that the Council on Chiropractic Education has laid out what he referred to as "the minimum requirements" for education in this field. This suggests that not enough is being done to enhance the education of chiropractic students with regard to musculoskeletal chest pain, and perhaps it is necessary to undertake a study across the chiropractic colleges.

Chiropractic colleges in North America tend to use chiropractic physicians as the main faculty in the diagnosis classes. Therefore, it is commonly the case that cardiology classes are taught by chiropractic physicians with expertise in family practice, such as those who have earned diplomate certification (advanced postgraduate training programs) in family practice and internal medicine. However, these programs should not be seen as equivalent to medical residencies in family practice. They usually require approximately 360 additional hours of didactic training, with only part of that training in a clinical setting. There are efforts underway to develop the 'advanced practice' chiropractic physician, with one chiropractic educational institution offering a training program coordinated by a medical physician. However, this is in its infancy and much needs to be worked out. This does indicate a growing level of interest in this kind of training, and will produce chiropractic physicians even better able to correctly differentiate a diagnosis of musculoskeletal chest pain. The participants in the focus group made no mention of postgraduate training or opportunity, yet this represents the one area beyond the standard curriculum where further training can be gained and is therefore the only way in which current chiropractors may finally gain new understanding of processes such as discussed here.

It would be worthwhile to survey the academic institutions to gain a better understanding of what is currently being taught across the chiropractic curriculum, as well as through postgraduate offerings. Focus groups comprised of diagnosis and cardiology instructors, postgraduate instructors, and representative field practitioners, can be performed. Such efforts would help derive a better picture of the current reality across the chiropractic profession with regard to education in musculoskeletal chest pain.

Interestingly, concern was also raised by participants that the current training in medical schools was inadequate, and that in part this was due to instructors who were not completely conversant with some of the more traditional means of diagnosis. An excerpt from the medical focus group comment underscores this observation: "... most cardiology now is very simple... Which test will give me the diagnosis? Most cardiologists...don't know auscultation... how to listen to the heart... professors for 15–20.... were never taught." This comment suggests that the 'low-tech' art of auscultation is being lost in medical practice, or increasingly replaced by 'higher-tech' laboratory testing in driving the process of diagnosis for cardiac problems, an opinion echoed in the literature [64,65]. A study of curricular and postgraduate medical education may similarly provide insights relative to current teaching and skills development for medical practitioners in managing musculoskeletal chest pain.

### Profession and health policy

As a professional issue, attention needs to be afforded the inherent uncertainty and complexity of chest pain diagnosis and the sometimes dynamic interplay between 'diagnosis' and 'treatment' whether in managing a given patient in actual practice or in attempting to define an appropriate evidence-based professional 'standard of care'. This is perhaps particularly true for a condition such as musculoskeletal chest pain, given the obvious dearth of established evidence from which the clinician may draw or on which to form definitive professional recommendations to guide current clinical practice. As a corollary example of an empirical 'treatment-based' diagnostic strategy, a presumptive diagnosis of gastroesophageal reflux disease (GERD) may be pragmatically validated in practice following a patient's favorable symptomatic response to a short course of prescriptive therapy such as proton-pump inhibitors, perhaps preempting or potentially avoiding more invasive or costly diagnostic tests such as endoscopy [66-72]. Non-cardiac chest pain, defined most simply as recurrent episodes of unexplained retrosternal pain in patients lacking a cardiac abnormality after a reasonable evaluation, is associated with repeated emergency room utilization [73] and may be treated empirically with antidepressants [74].

We might ask whether there is a role for musculoskeletal assessments within clinical chest pain diagnostic algorithms that is not being fully exploited in current practice, particularly given the relative costs and safety of the more invasive and resource-intensive alternatives, not to mention patient preferences. In cases where an early 'low-tech' assessment offers a presumptive suggestion that chest pain may be musculoskeletal in nature, might a short course of manual therapy help to validate a presumptive diagnosis and guide treatment decisions [75-80]? How much, and what level, of current available evidence is needed to support clinical decisions or professional recommendations that favor low-technology, low-cost noninvasive procedures early in the diagnostic workup, or that justify manual therapy following an empirical validation of a presumptive musculoskeletal diagnosis? These are tough questions with no easy answers, especially given the inherent high-risk, uncertainty, and complexity of chest pain diagnosis, and the possibility that chest pain may present with any mix of multiple musculoskeletal or non-musculoskeletal etiologies or comorbidities.

Musculoskeletal chest pain as an area of inquiry fits well within the health services research agendas outlined in health policy initiatives to improve primary care, patient safety, and the delivery of evidence-based cost-effective care. As identified earlier, the appropriate management of chest pain raises a host of considerations relative to improving cross-disciplinary coordination of care within the health care system, whether for diagnostic consult, referral for treatment, or continuity of care in the overall management of the patient's total care plan. The potential implications for improving patient safety are also worth noting, specifically relative to enhancing prompt accurate diagnosis, and where possible decreasing unnecessary exposure of patients to higher risk or more invasive procedures. As a specific target within the primary care, patient safety, and cost-of-care initiatives, 'ambulatory-care sensitive conditions' are identified as those conditions that, when managed appropriately in the outpatient setting, can prevent unnecessary and costly inpatient care. Chest pain is high on the list of high prevalence ambulatory conditions associated with 'avoidable hospitalizations', and with repeated high-cost hospital emergency room utilization [73]. While discipline-specific approaches to diagnosing or treating non-cardiac chest pain of gastrointestinal, psychiatric, or musculoskeletal origin have served useful, the quality and safety of patient care may be even better served by a coordinated cross-disciplinary research effort and practice approach.

A final health policy consideration relative to health workforce planning and development, is in acknowledging that chiropractors serve a role as a first point of contact with the health care system or as the main source of care for many patients, particularly in rural or medically underserved areas [62,63]. Policies to improve access to care by promoting the primacy of the relationship between usual-source practitioners and their patients, must also pay due attention to developing the role and requisite skills of non-medical practitioners such as chiropractors to appropriately manage or co-manage a broad range of conditions such as chest pain in primary care settings.

## Conclusion

Our research leads us to offer a number of recommendations for practice, research, education, and policy. Certainly, the investigators and members of the focus group feel that more education should be required in the diagnosis and management of chest pain. Research is also needed about the educational opportunities and challenges revolving around interdisciplinary care and practice.

Greater outreach to the medical research community, and indeed to the wider medical community, will help to enhance skill sets and collaborative opportunities. This outreach may help to drive research in those areas where it is most needed: diagnosis, incidence/prevalence, treatment, and clinical protocols within and across disciplines. By developing a research base, it will be possible to establish appropriate standards for care, and these can be enhanced by creating multidisciplinary panels to explicitly improve cross-disciplinary coordination of care.

## Competing interests

The author(s) declare they have no competing interests.

## Authors' contributions

Monica Smith conceived the study, and coordinated the focus group meetings as well as performed thematic analysis of transcripts and helped write the manuscript. Dana Lawrence performed thematic analysis and coding of transcripts and prepared components of the manuscript. Robert Rowell also performed thematic analysis and coding of transcripts and prepared components of the manuscript. All three authors read and approved the final manuscript.

## Supplementary Material

Additional File 1 Focus Group Questions for MD & DC Chest Pain Study. Click here for file

Additional File 2 Seminal excerpts of dialogue from focus group transcripts, bytopic. Click here for file
