# Supplementary material for: Management of chest pain: exploring the views and experiences of chiropractors and medical practitioners in a focus group interview
Source: Chiropr Osteopat. 2005 Sep 2;13:18. doi: 10.1186/1746-1340-13-18 (PMC1236944; doi:10.1186/1746-1340-13-18)
Supplement: Additional File 1 — Focus Group Questions for MD & DC Chest Pain Study. [file 1746-1340-13-18-S1.pdf]

ADDITIONAL FILE 1: Focus Group Questions for MD & DC Chest Pain Study

1. Many patients today utilize the services of both MDs and DCs. What kind of information do these patients share with you regarding their experiences with other doctors? What type of advice or information do you share with these patients regarding their experiences with doctors from other fields? What have been your positive and negative experiences in fostering interprofessional relationships between MDs and DCs?

2. For what clinical reasons would a chiropractor refer a patient with chest pain to an MD? For what clinical reasons should a patient with chest pain be referred to an office-based MD generalist vs. an MD specialist vs. emergency medical services?

3. When a chiropractor refers a patient with chest pain for medical care, what type of information should accompany that referral? Should the MD send follow-up clinical information to the chiropractor following a referral, and if so, what type of information?

4. In your experience, do MDs ever suggest that a patient try chiropractic care for chest pain? If so, are MDs more likely to **formally refer** a patient to a specific DC, or to just generally **informally recommend** that the patient might try chiropractic?

5. For what clinical reasons would a medical doctor recommend or formally refer a patient with chest pain to a chiropractor? If an MD refers a patient with chest pain to a chiropractor, what type of information should accompany that referral? Should the chiropractor send follow-up clinical information to the MD following a referral, and if so, what type of information?

6. As a provider what, if anything, would you like to see change in the relationships between chiropractors and medical physicians, specifically how should formal patient referrals and the exchange of patient information take place? Could "best practices" for coordinating the care of chest pain patients between MD and CHIROPRACTICproviders be identified or developed? Are there specific clinical or research questions that could help inform the development of "best practices"? What methods might be used to gather information to answer those questions?

7. In preparation for this focus group, you received a selection of clinical and research literature on the topic of **musculoskeletal chest pain**. Based on this literature and also drawing from your own clinical experience, what specific clinical or research questions about this condition need to be answered? In particular, would it be useful and informative to study the prevalence and current clinical care of chest pain presenters in ambulatory settings? Is there potential value to incorporating musculoskeletal diagnostic procedures into overall chest pain evaluation protocols, and is this feasible? What research methods might be used to begin answering these questions, and is it feasible to conduct or coordinate a multidisciplinary research effort along this line of inquiry?
