# Supplementary material for: Management of chest pain: exploring the views and experiences of chiropractors and medical practitioners in a focus group interview
Source: Chiropr Osteopat. 2005 Sep 2;13:18. doi: 10.1186/1746-1340-13-18 (PMC1236944; doi:10.1186/1746-1340-13-18)
Supplement: Additional File 2 — Seminal excerpts of dialogue from focus group transcripts, bytopic. [file 1746-1340-13-18-S2.pdf]

APPENDIX TWO
Seminal Excerpts of Dialogue from Focus Group Transcripts, by Topic

Topic:
(1) Diagnosis and prevalence
(2) Treatment and prognosis
(3) Complexity (chest pain as a chronic, multifactorial, or comorbid condition)
(4) Inter-professional coordination of care
(5) Best practices and standardization of care
(6) Training/Education

Topics: Diagnosis, Interprofessional Coordination

*MD2***:** "…[for] a patient [with] a chest-related complaint or a thoracic-related spine complaint…[we] ask have they been treated by anyone else and who and what kinds of treatments or medications have they had and the last time they were seen and so forth and how did they respond….[i.e.] Who else have you seen? What have you had done? What did you perceive as far as being positive or negative? How does that factor in as far as why you're at my office today, and so forth?..."
*MD1***: "**I try to get as full of a history as possible with the patient - and that would include any and all visits with previous physicians - I try to focus initially on the chief complaint to start with and then get a full history following that. I also try to obtain previous medical records from all health care providers to that particular individual, and if I don't have that before we see the patient then we try to get it either during, by fax, or afterwards. Generally speaking, patients are quite open with their discussions and their experiences with previous health providers. I have not experienced a problem obtaining the history. They are quite aware that everything they say in the office is strictly confidential so that makes it fairly simple. Problems that I've had at least from a history standpoint involve memory more than anything else…and that's why it's good to get written material if possible…"
*DC1***:** "…[in] practice…We do a good history on our patients… ask the other doc if he could just fax me the reports on any studies that he's done and his treatment plan….Twenty years ago this was a big problem because we couldn't get any reports. They wouldn't send us anything and the patients were afraid to tell either one that they were going to the other doc. I don't see any of that anymore. It [barriers to interprofessional communication] really has broken down these last 20 years…where [now] I get on the phone and call the doc and say*, what are you doing? Is there anything contra-indicated for me to doing this?"...* It's no problem…".

TOPIC: Coordination of care
*MD1***: …**I have had a number of referrals [from DCs]. Those referrals have been excellent referrals. Certainly justifiable referrals with disclosure, full information. I guess the question in my own mind is how prevalent is that? "

TOPIC: Coordination of care
*MD1***:**[Need for sharing information] varies significantly. And it also varies from patient to patient and what their actual problem is.

TOPIC: Coordination of Care
DC1: **"…**for emergency medical I would say if the person's in distress: chest pain, sweating, pressure, you know based on the symptoms either a pending or an active MI. Those go from my office to ER right away." *MD1*: **"** They go from our office to the ER also."

TOPICs: Diagnosis, Treatment, Coordination of Care
DC1: …[I would not think to refer]….If I can put my finger on it and cause pain literally, I will attempt treatment at that point. Especially if vitals are good, there are no activity problems, if the patient has no shortness of breath, none of your classic findings, but they still have chest pain and I can put my finger on it and find that it's possibly a sternal costal junction irritation, I'll treat it. But I do rule out all the other possibilities. So they have to be free of the other indicators before I won't refer them." *MD1*:**"**Okay, so basic good history."
DC1:**"**Yeah, that's it."

TOPICs: Diagnosis, Coordination
DC2: **"**And I don't think I'm unusual in that I seem to see a lot of patients who have costal chondral origin pains. I don't know what percentage of them in my practice. Most of what I see is low back, obviously. But I see a lot of these patients [with musculoskeletal chest pain]. And it hurts right here and when you put a hand on it you can ice it and improve it. In those cases then I won't let the patient out unless I have some of these other issues with it. But when you see someone who is 20-years-old and they have a tender spot on the chest wall, I feel pretty comfortable with that". *MD1*: **"**We do see a number of those as well, so we end up referring those out". *DC2*: **"**Where do you send those?" *MD1*: **"**Most of the patients we see are by referral, so they're sent back to their original doc." *MD1*: "…[for patients we classify as]… Chest wall or musculoskeletal… sometimes we'll do some limited study whether it's an EKG. Rarely, but if it's still undecided we'll go on to have some type of stress test."

TOPICs: Diagnosis, Chest pain as complex condition, Coordination, Training
MD1: **"** There is no question that one can have both problems [of musculoskeletal and non-musculoskeletal chest pain] and that's where life gets more interesting."
DC1:"[for patients with both musculoskeletal and non-musculoskeletal chest pain], **c**oncurrent care is fine then." *MD1*: Yes, as long as you make appropriate diagnosis of both conditions and that sometimes can become troubling. I did see a patient with chest pain that was being treated by a chiropractor and received several weeks of manipulations. I couldn't find any tenderness anywhere and I thought that that patient had typical angina type of symptoms. I recommended a coronary angiogram and because he felt that he was receiving appropriate care even the symptoms never disappeared, it was extremely difficult to convince him that he needed an angiogram. He sought a second opinion and miraculously he agreed with me. But he ended up having an angiogram and had severe three-vessel disease. That was the only I don't know if I want to call it a negative experience. It wasn't a referral. He just happened to have gone to both. I was concerned that it wasn't picked up by the chiropractor. I thought it should have been. I have that with generalists and family practitioners too." *DC1*: **"**Everybody gets busy. That doesn't okay it." *MD1*: **"…**it's extremely important that all of us whether we're specialists or otherwise consider ourselves as basic primary care physicians no matter what we do and take a good history."

DC2: **"…**I find frequently patients deciding what information the chiropractor needs to know about "my low back." I need to tell him about my low back pain, you know this constipation that I've had I don't really want to talk about it. And so it behooves us to really draw information out of our patients." *DC1*: **"**That goes right back to doing a good history. You do the review of systems. You force it out. *MD1*: **"**And I'm not sure if MDs understand that chiropractors do a history. That's part of the information that they need to know. *MD-DC*: "[chiropractic education needs to emphasize that] the case history is very essential for doing a good diagnosis, it has been shown in most specialties that if you really do a good case history you have a very normal link concerning the diagnostic process. *MD1*: **"**Well, it goes back to medical school, 90%."

TOPICs: Diagnosis, Complexity
MD-DC: **"**But about the [chest wall] tenderness and the direct tenderness spot and as you say reproducing the tenderness, I mean that's not enough because you see it both as coincidental finding." *MD1*: **"**And many times they will describe two different types of chest pain." *MD-DC*: **"**Yes, so if you forget to do a proper case history and do an estimate then you might be bad off." *DC1*: **"**Yes, there's nothing that we do that would be done in lieu of a history. It's in conjunction with. We actually do a little more I think palpating than the normal GP, but it should never be done in lieu of that case history or physical. It's just in conjunction with. There's no reason I'd see why we would ever limit our history taking or our physicals."

TOPICs: Coordination, Standards
MD-DC: **"…**with the different specialists, doctors, DCs…[some may prefer more information than others]". *MD1*: **"**Sometimes you need it. Sometimes you don't. That's the problem." *DC2*: "The way I look at if I'm sending a patient to you, for example, or any other physician, if I send all of my exam and history findings they're going to do their own history and exam anyway, so that's why I would send like a paragraph *here's what's going on* and ask them questions more than anything." *MD2*: **"**The exception to that is if I send patients to a neurologist, for example, they are specifically concerned about certain things and I'm ordering like an MRI for a neurological consultation." *MD1*: **"**But with a consultation from us (cardiology) you'll get a fairly hefty report back, which will include the history and physical and everything else. We just crank them out in a standard kind of way just to make sure that we cover all points kind of like you talk about review assistance we do routinely. It depends what you want back." *DC2*: **"**I guess I would prefer to get that back." *MD1*: **"**You can skip the first three pages and just go to the bottom line." *DC3*: **"**I think early on I used to probably send more out than I do now because I wanted to open up that line of communication." *DC1*: **"**We did that more educationally so the doc knew *hey we did do a physical*." *DC3*: **"**Anymore, though, [having been in practice awhile], it's cut to the chase." *MD1*: **"**Yeah. *This is the problem. Help us out".*

TOPICs: Diagnosis, Treatment, Complexity, Coordination
MD2:We're topped down. Unless it's obvious that somebody has a GI cause or a pulmonary cause of chest pain… So the differential diagnosis, well #1 it's cardiac, so we see them first. So usually they haven't had any other workup. *Make sure the heart is or isn't the problem.* So we see them and do our EKG stress test. You know, in our practice, and I think I can speak for many cardiologists, we see them, I do my little bit, and unless …it's just blatant (pulmonary or

GI, for example) and hits me in the head, I'm going to say, *alright, I have my blinders on. The heart's okay…. Go back to your family physician."*
MD1: **"**If we don't [refer the patient back to their primary physician], we're not going to see any more referrals….. Now we've done it on occasion. If you know the referring physician well enough that he says, *Sure whatever you think. Just go for it.* Now there's been an occasional patient or two where I say, *boy, you know this is kind of obvious. We need a GI workup*, but then I'll at least call [the primary care doc] and say, *hey listen, this sounds more GI. Do you want me to make a call?* So that's the way we operate now and I think that's the way we need to operate."
MD2: I think the primary care docs when they get these patients back and they do the GI and pulmonary…the musculoskeletal evaluation they do their little bit as to what they know and if they can't take care of it, I would imagine that the majority of patients on their own seek chiropractic as an option. I would imagine that the majority of the allopathic or even osteopathic physicians don't rush to send people to chiropractic medicine just because we're not taught, we don't know. It's that set reflex that you kind 'a have to train almost.
MD2: Yeah, but when people do come to us, and I think to the family docs too, I would agree with you. Now, compared to maybe 20 years ago, [patients are saying] *I'm seeing a chiropractor. Is that okay? Yeah, of course. Is he helping? Yeah. That's great. You've seen him.* But you know, I think most doctors, most MDs or DOs would say *yeah, that's right. Keep going. Give it a good shot.*
MD1: **"**I think the only thing we do set aside from agreeing with going to a chiropractor and that is just to make sure that in our opinion there is not a cardiac issue and once we've ruled that out, whatever treatment modality could potentially help is to my mind the correct one."
MD1: Very often I certainly put down in my diagnosis - in fact I have this week - that this is clearly a musculoskeletal issue and the question then is how best to treat it. And if there is clear cut evidence that chiropractic modality in treatment is more effective than non-steroidal, which has not been verified in an evidence-based manner then that's the way it should be. The question then is who makes that decision. Could it be a suggestion? Sure. Why not? Now, could one influence the decision [by the primary care doc]? I suppose. You could certainly give a list of treatment options. But I'm not sure that's our job either. Once it's non-cardiac basically we're fired if you look at it. Unless [the specialist] is also the primary and that does happen rarely, but it could so…"
MD1: **"**Perhaps once you clarify treatment of this particular problem - and it's a fairly widespread problem - musculoskeletal chest pain is pretty common. I saw some quotes [in the literature] as 20-30% and that's probably true. I certainly think in my (cardiology) practice that's probably close to what I see and that's a huge number of patients. I would first try to get better information, more information, and disseminate it because if there is a better way of treatment then that's the way we should all go. And then I would direct it to primary care physicians and the emergency room. It's a huge area that sees these types of problems and that would be a great place for triaging."

TOPICs: Treatment, Complexity
DC1: **"We** used to 20 years ago, [have patients] walking into our office saying, *hey, everyone's throwing me out. Nobody believes me. I'm in pain.* And we used to see them. And in fact, back then, 25 years ago they actually used to be at that point in time referred to a psychiatrist for pain control. You know, maybe you're magnifying your problems a little bit.

Well, that has really reduced quite a bit and they are utilizing [chiropractic] a little more at this point.

*MD-DC***:** But again one more point should probably be that [timely] referral from an MD to a DC to actually prevent it from becoming a chronic pain condition. As you said, you have a lot of patients coming in for a second, third, fourth opinion and they take up a lot of resources and they're not easy patients. And they're not actually the ones you want to deal with. So if those pain conditions could be stopped, theoretically, early on, then everyone could be happier. We don't like to treat those chronic patients who are absolutely focused on pain where it really doesn't make a difference. They're not easy. *MD1***:** Yeah, you're right. The question then comes when is it a chronic problem and at what point do you refer? If it could be done earlier, yes it would make sense to me. Very often they end up going through all of these modalities. Maybe they don't need to. And perhaps the order is incorrect. Maybe they shouldn't be getting injections and steroids all over their chest. Maybe they ought to go to a chiropractor first and then if they're not successful maybe then see the other one. It might be that putting a needle in someone's chest is not the best approach either." *DC1***: "**Some of the new literature… is showing pretty good indications that early manipulation treatment might eliminate some of this chronicity that we're seeing with these musculoskeletal problems."

*MD1***:** Of course, the question is, is this a homogenous problem to begin with? It may not be. It could be multiple etiologies. And none of us still understand Tietze's diseases or costochondritis or whatever you want to call it. We don't know what the etiology is. Without knowing the etiology, in my opinion, it's difficult to treat it. We don't know how to approach it.

TOPICs: Standards, Complexity

*MD2***:** For a specific entity, such as musculoskeletal chest pain, Cardiologists would not be the pegs in the "best practice" model. It would have to be the primary care internist….and for best practice models you have to have outcomes based data. So in order to get outcomes based data you have to get everybody from top down on the same chain… the younger more recently graduates may be more apt to give it a shot. Why not? It's going to help the patient…. And you need objective data. You can't just have subjective on this. You have to show the old guard, the "iffy" guard and the young guard, how we did it objectively and here's your data."

*MD1***:** It happens on many levels in cardiology. Many times we're treating sometimes by the seat our pants and not by clinical evidence and when the clinical evidence is out there… a huge area, for instance, was the treatment of arrhythmias. Premature ventricular contractions we all felt were just *bad* things. But we found these wonderful drugs that could abolish them. When you did a clinical trial investigating arrhythmias study - a CASS trial - we ended up showing that mortality actually increased with therapy. We were not doing the right thing. Well, it was a little drastic, but it certainly changed the way PVCs were treated. They just weren't treated anymore. We were not doing anyone a favor. Yeah, it's got to be basic black and white evidence and that's where it's gotta come from. And if it's out there then no-one has a choice. It's there and that's the way you gotta go. And if you can show that you have a significant patient population that can benefit from chiropractic therapy and that it's safer for the patient then it's a simple decision, I think.

*MD2***:** The chest pain patient that's 40 to 60-year-old, I don't think it's that hard to figure out what's going on with the majority of them. It's the person between 60 and 85 that have musculoskeletal pain, who has calcification of everything in the thorax and they do have

coronary disease….by then they have a little achalasial or esophageal dilatation and…their smoking history finally caught up with them and the lungs are flared up. So they have multi-factoral causes for chest pain. So allopathic medicine or even osteopathic medicine may do their part, but you may still have 16 or 20% of patients who are not going to die but they're still having pain. *MD1*: Yeah. I have a bunch of those.

TOPICs: Diagnosis, Standards
DC1: [For chest pain, are musculoskeletal exams] already done in a GP's office? *MD-DC*: Nope. Well, at least that's my experience. *DC1*: **I**t is in my office. I have to do both. That's maybe because my primary is musculoskeletal and that's what I'm looking for, but I also have to rule out all the possible reasons for that chest pain. *MD1*: Musculoskeletal…I check everybody's sternum for tenderness routinely including MI patients in the hospital. And oftentimes I'll find chest tenderness even with an MI situation. That's part of my routine physical. *DC1*: Was that taught culture? Then it's already there. *MD1*: Should be. Whether it's being done I can't answer it. *MD2*: No question. It's not being done…. *MD1*: Probably not. *MD2*: … cause you know 8% of my ER chest pains… *MD1*: I was just going to mention the ER. *Hey, did you see the patients?* *MD2*: Yeah. Exactly. *MD1*: Can't just wave at them. But that's a different problem. *MD-DC*: "…[even with] a thorough examination of the complete musculoskeletal system… and… a lot of musculoskeletal components all over the chest wall…[may also have cardiac]… So it's very complicated actually. *DC1*: So wouldn't that make sense?... You can have two separate problems. *MD-DC*: Yes. But what I hear is that sometimes you feel very confident that if you have a tenderness and that's a musculoskeletal cause on it's own, but I'm not that comfortable all of the time that that is the case…. Because if you actually do a full cardiological workup and a full musculoskeletal exam it overlaps and that's when it becomes difficult.

TOPICs: Diagnosis, Treatment, Complexity, Standards, Training
MD-DC: When do you decide what to do [in your differential workup]? *MD2*: It depends on if it's pretty classic and you have the diagnosis for the history which you do in many instances and then it's just a question if it's something that's severe or is it something that you can do a noninvasive testing on. People who have unstable symptoms who are accelerating, getting worse, you do an angiography. There's no need to do a stress test on those patients. But in general I think how we do things may be different than how the rest of the cardiology community does things in [our local community] even, but I think that we're taught… *MD1*: Well, there's ACC (American College of Cardiology) guidelines… *MD2*: Yeah. They're guidelines where you show there are objective items of ischemia before you take the stable symptoms…(talking over one another) *MD1*: That's the recommendation. *MD-DC*: Yeah. Because we don't have gold standards for classifying musculoskeletal conditions…or [how to] actually rule out that it is or it is not. *MD1*: It would be nice to have some type of procedure, test or whatever that would clearly show….. I do agree that musculoskeletal conditions exist in conjunction with coronary artery disease and we do see that commonly…. Again, we don't know the exact etiology of that and that's still… *MD-DC*: Do you look at the back? *MD1*: Do I look at the back? Part of my general physical is to at least push on the back, yes. But I'm an old timer. *MD2*: I was taught by old timers. I don't push on the back unless there's a reason to. But most cardiology now is very simple. Which test will give me that diagnosis. Most cardiologists wouldn't - I'm quoting this from a publication - about 34% of cardiologists

don't know [auscultation?]. We had a course specifically we started in our fellowship how to listen to the heart. I mean it sounds stupid, but you'd be amazed at professors for 15-20 years come. They were never taught. *DC1*: How does that happen? *MD-DC*: You talk about standardized procedures. *MD2*: How does an illiterate kid graduate from high school? It's the same thing, that same ‘somebody else will take care of it kind of concept’. *DC1*: That's exactly right. *MD1*: I spend more time pointing out -------4 split heart sound. I'm happy when [medical students] know what a murmur is, but uh, that's half of what we do here. *DC1*: Or is it because technology does it? *MD1*: Yeah, that's a big part of it. Yeah, no problem we'll do an echo. That'll tell me what's leaking. There's a murmur. That's all we need to know. That's why I tell the students, *You tell that echo tech what valve is leaking and what valve we're looking for*. Just confirming. *DC1*: … students… want the easy route. They don't want to have to learn it to the level that we think they should. *MD1*: Well…[and the mentality of not wanting to] sit there and think what does this patient have and why? Let's see how many tests can I order and how quickly and then I'll wait for the answers.
